# Promoting physical activity and a healthy active lifestyle in community-dwelling older adults: a design thinking approach for the development of a mobile health application

**DOI:** 10.3389/fpubh.2023.1280941

**Published:** 2023-11-29

**Authors:** Kim Daniels, Ryanne Lemmens, Els Knippenberg, Nastasia Marinus, Sharona Vonck, Jan Baerts, Jochen Bergs, Annemie Spooren, Dominique Hansen, Bruno Bonnechère

**Affiliations:** ^1^Department of PXL – Healthcare, PXL University of Applied Sciences and Arts, Hasselt, Belgium; ^2^REVAL Rehabilitation Research Center, Faculty of Rehabilitation Sciences, Hasselt University, Diepenbeek, Belgium; ^3^Department of Digital, PXL University College of Applied Sciences and Arts, Hasselt, Belgium; ^4^THINK3 Simulation & Innovation Lab, Faculty of Medicine and Life Sciences, Hasselt University, Diepenbeek, Belgium; ^5^Technology-Supported and Data-Driven Rehabilitation, Data Sciences Institute, Hasselt University, Diepenbeek, Belgium

**Keywords:** design thinking method, digital technology, physical activity, older adults, mobile application, mHealth, motivation, participatory design

## Abstract

**Background:**

Physical activity (PA) has wide-ranging, and well documented benefits for older adults, encompassing physical, cognitive, and mental well-being. The World Health Organization advocates for a minimum of 150–300 min of moderate intensity PA per week, supplemented by muscle-strengthening exercises. However, the rates of PA among older adults remain a concern. While portable technologies hold promises in promoting PA, sustaining long-term engagement continues to be a challenge.

**Objective:**

The aims of this study are to identify barriers and facilitators to PA in older adults, to develop an mHealth app promoting PA and an active healthy lifestyle in collaboration with community-dwelling older adults guided by the design thinking process, and to test it.

**Methods:**

A co-creative process was used, employing design thinking. Interviews were conducted to understand the needs of the target population and identify the problem of insufficient PA. Two cocreation sessions involving older adults and experts were conducted to generate innovative ideas. Participants were selected based on age (≥65 years), no severe illness, Dutch language proficiency, and active participation ability. Results were qualitatively analyzed and coded. Finally a prototype was developed and tested.

**Results:**

Interviews with older adults highlighted diverse perceptions of PA but unanimous agreement on its importance. They recognized health benefits such as improved mobility, balance, and reduced fall risk, while emphasizing the social and mental aspects. Barriers included poor health, time constraints, weather conditions and fear of falling. Cocreation sessions identified key topics: perception of a healthy lifestyle, coping strategies, mHealth App features, screen visualization, and tailored notifications, which led to the development of a mobile app promoting PA and an active lifestyle. The app was stepwise prototyped.

**Conclusion:**

This study emphasizes the importance of promoting PA among older adults through a collaborative design thinking approach. However, the implementation of mHealth apps faces obstacles due to the digital divide, necessitating personalized solutions to bridge the gap. Moreover, it calls for further research to investigate the long-term impact of such interventions and explore behavior change patterns in this population.

## 1 Introduction

Regular physical activity (PA) is important to improve wellbeing in the overall population and particularly in older adults. According to the World Health Organization (WHO), the number of people aged over 65 years old will increase significantly to 2.1 billion in 2050 (1). Given the unprecedented rate of the population aging, it is of the utmost importance to promote regular PA in older adults to try to tackle this huge public health challenge.

Research has unequivocally demonstrated wide-ranging, and well-documented health benefits of regular PA on physical, cognitive and mental health even at an older age (2–6). Based on evidence, the WHO recommends that individuals aged 65 and above should engage in at least 150 min moderate-intensity aerobic PA, or at least 75 min of vigorous-intensity aerobic PA weekly, supplemented with muscle-strengthening activities at least 2 days per week and balance or multi-component exercises (7).

To achieve a moderate intensity, exercise should be performed between the first and second ventilatory threshold, while vigorous exercise is typically performed above the second ventilatory threshold. Determining the appropriate exercise intensities can be facilitated by the addition of the subjective perception of exertion experienced during exercise (8–10). Different methods can be used to evaluate this perception of exertion such as the Borg’s rating of perceived exertion (RPE) scale (11, 12). A more easy and straightforward alternative is the talk-test, which involves assessing exercise intensity by the level of difficulty in carrying on a conversation (13, 14). When ventilation becomes sufficiently elevated to make conversation somewhat difficult, this is near the second ventilatory threshold and is considered moderate intensity. Above this level, where conversation becomes challenging, is considered vigorous intensity (15).

However, despite an ever-increasing awareness of these recommendations and benefits rates of participation in PA in older adults remain a substantial concern, with many failing to meet the minimum recommendations set by the WHO (7). The global prevalence of physical inactivity is estimated to be around 31% (16, 17). Older adults have a more sedentary lifestyle compared to younger adults, spending more than 9.4 h in sedentary activities per waking day. The absence of enough PA, whether combined with other modifiable risk factors like smoking and alcohol consumption or not, significantly raises the likelihood of various health complications (18). A recent meta-analysis emphasized that physical inactivity may lead to a higher mortality rate in high-income countries compared to hypertension, diabetes, or smoking (19, 20).

To address the lack of PA and insufficient awareness of its benefits among older adults, mobile Health applications (mHealth app) have emerged as promising and cost-effective tools (21–24). The availability and accessibility of smartphones and tablets has led to an exponential increase in the number of interventions delivered through mHealth (25). Regrettably, interventions targeting the promotion of PA among community-dwelling older adults often struggle to maintain long-term engagement (26). Hence, gaining a comprehensive understanding of the obstacles and enablers to the acceptance and adherence of mobile health (mHealth) for PA in older adults is of utmost importance.

The active engagement of end-users in the creation and development of interventions has the potential to significantly enhance their sense of ownership and promote a better understanding of their authentic needs and desires. Human-centered design thinking methodologies have demonstrated efficacy in generating valuable outcomes by collecting end-users’ thoughts, needs, motivation and attitudes. It is a systematic co-creative process of innovation that focuses (in several phases) on cultivating deep empathy towards end-users, understanding their desires, needs, and challenges to gain a comprehensive understanding of a problem with the goal of creating more effective and comprehensive solutions (27, 28).

By engaging the target population as co-designers and stakeholders in the design thinking process of the mHealth app, a deeper understanding of their requirements is gained. This hopefully results in a better user experience, usability and user acceptance, thereby creating long-term impact and engagement (29, 30).

Despite its potential, incorporating end-users as a partner in designing health technology is an understudied area of research (31), especially with older adults, as most studies rather focus on testing prototypes and their feasibility (32–34). Moreover, collecting validated evidence on the value of the involvement of the end-users in the design thinking process is difficult, as its vague processes are considered incongruent with commonly used research methods for outcome measurements (35). One of the reasons is that the design thinking process is rarely described in research papers.

Therefore, the objectives of this study were threefold: 1) to identify barriers and facilitators to PA in older adults, 2) to develop an mHealth app promoting PA and a active healthy lifestyle in collaboration with community-dwelling older adults guided by the design thinking process (36), and 3) to test it.

## 2 Materials and methods

This study was registered at Clinical Trials.gov (NCT05650515) and at OSF Registries (DOI 10.17605/OSF.IO/MA7EW), and was approved by the Ethical Committee of Hasselt University (B1152023000011) and a written informed consent was obtained from all subjects before their participation.

**Figure 1:**
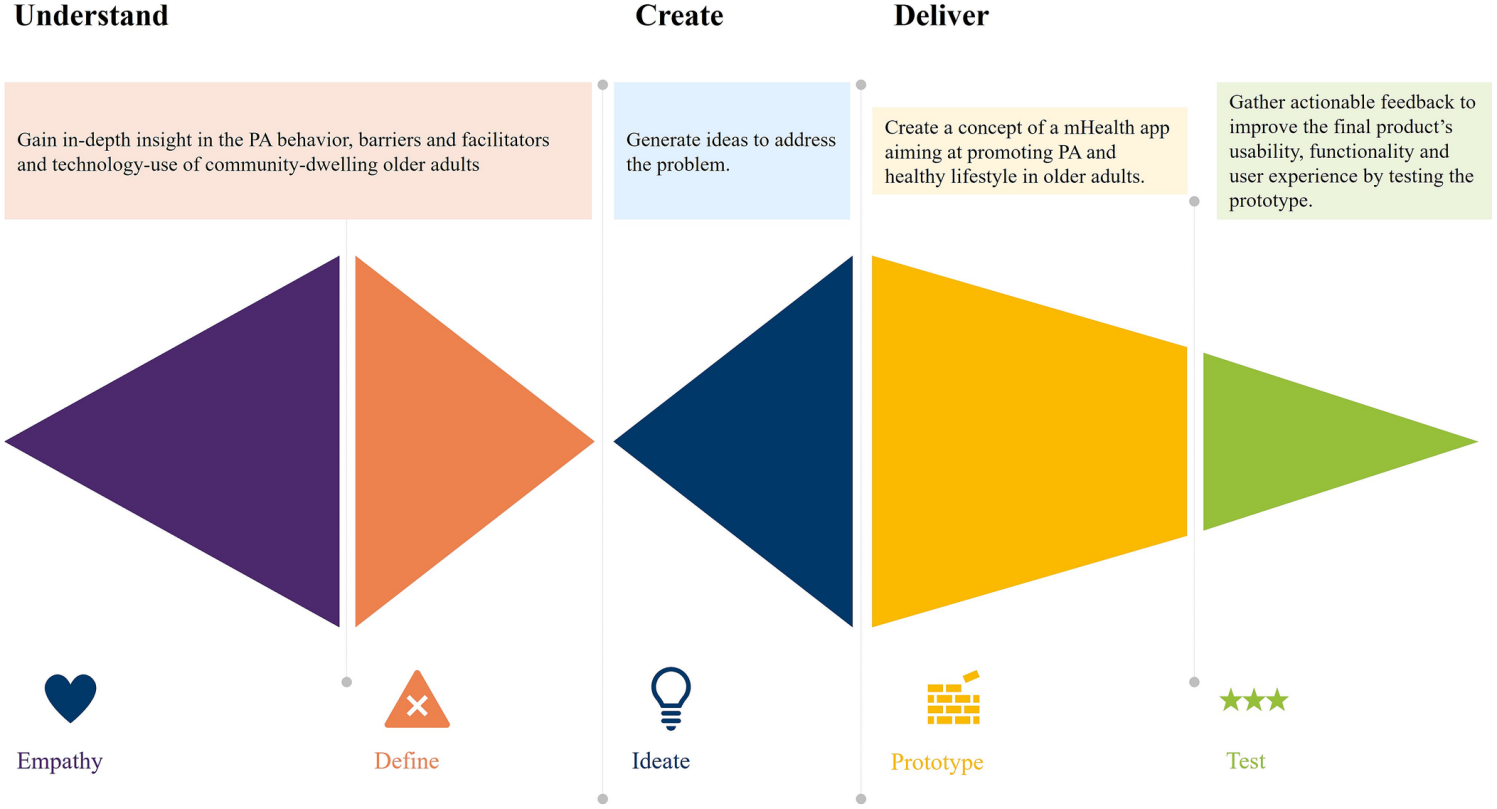
Phases of the design thinking process applied within this study.

To gain insight into the needs of older adults, a co-creative process was applied based on the design thinking method (Figure 1) (23, 27). In depth – interviews were conducted to grasp the target populations’ needs and motivations and better understand the problem of limited PA. Furthermore, two co-creative workshops were subsequently conducted (one with exclusively older adults and another with a combination of older adults and domain experts) to generate ideas that address the problem. Finally, a prototype was developed and explored on a small sample of the target population. This approach facilitated the iterative and user-centered design process that effectively tackled the complex needs of older adults in relation to PA with the support of technology.

### 2.1 Participants

For all phases, the inclusion criteria were: a) Community-dwelling older adults (≥65 years), b) no severe illness (e.g., no history of adverse cardiovascular events, cancer, diabetes mellitus, cognitive decline, stroke, Parkinson’s disease, multiple sclerosis), c) good understanding of the Dutch language and d) being able to actively participate in interviews, workshops, and pilot testing.

Interviewees were recruited via pitches at several senior citizen organizations. For the workshops, older adults, who had expressed interest in the project as participant of the interviews and met the inclusion criteria, were recruited. To create a varied group of older adults, participants were selected by using a convenience sampling approach. Domain experts in the second cocreation workshop were selected based on their expertise in the field of PA, technology and gerontology.

For the initial prototype testing, a cohort of 65 eligible participants meeting the inclusion criteria was recruited at the sport day event for the population of older adults of the city Hasselt, Belgium. Participants for the sport event were recruited through various means, including advertisement, social media, and word to mouth.

Socio demographic characteristics of the participants included in each project phase are presented in Table 1.

**Table 1:** Participants characteristics per phase.

Phase	#	Sex		Age	Exercise Habits					Experience with technology – use				
	N	F	M	Mean ± SD	150 min MVPA	75 min MVPA	Strength Training	Balance Training	no PA	Mobile phone	Smart-phone	Tablet	Computer	Smart-watch
Empathy and defining phase: In-depth interviews	22	14	8	76.05 ± 6.27	37.5%	43.75%	31.25%	6.25%	25%	17.65%	88.24%	35.29%	82.35%	0%
Ideate and prototyping phase: Co-creative workshops	16	6	10	68.33 ± 2.77	58.33%	16.6%	41.6%	33.33%	16.6%	6.25%	93.75%	56.25%	87.5%	0%
	6	5	1	72.33% ±5.73	0%	50%	33.33%	16.66%	33.33%	16.6%	83.33%	50.00%	66.67%	0%
Initial prototype testing of the alpha version	65	56	9	71.16 ± 4.33	Frequency PA per week (Mean ± SD)		Minutes MVPA per week (Mean ± SD)			27.69%	78.46%	50.77%	76.92%	24.62%
					3.69 ± 1.69		68.00 ± 40.30							

MVPA is moderate to vigorous physical activity.

### 2.2 Design and analysis

#### 2.2.1 Empathy and defining phase: in-depth interviews

To determine perceptions, barriers and facilitators of regularly engaging in PA, semi-structured, one-on-one interviews, lasting between 40 to 85 min, were conducted (and audio-recorded) between April and September 2022 by two researchers experienced in qualitative research. The interviews followed an Interview Guide designed to encompass relevant topics concerning PA (habits, intentions, obstacles and enablers), the use of technology and possible solutions for the lack of PA (the guide is presented in Supplementary Appendix S1).

The interviews were audio-recorded and further processed via thematic analysis, based on the guidelines from Braun (37). Data were described, summarized, and then interpreted in relation to broader implications. Descriptive codes based on patterns within the data were collated with a predominant focus on the identification of salient themes across the questionnaire responses. These themes were discussed with a second researcher, revised, and validated by all members of the team.

#### 2.2.2 Ideate and prototyping phase: co-creative workshops

**Figure 2:**
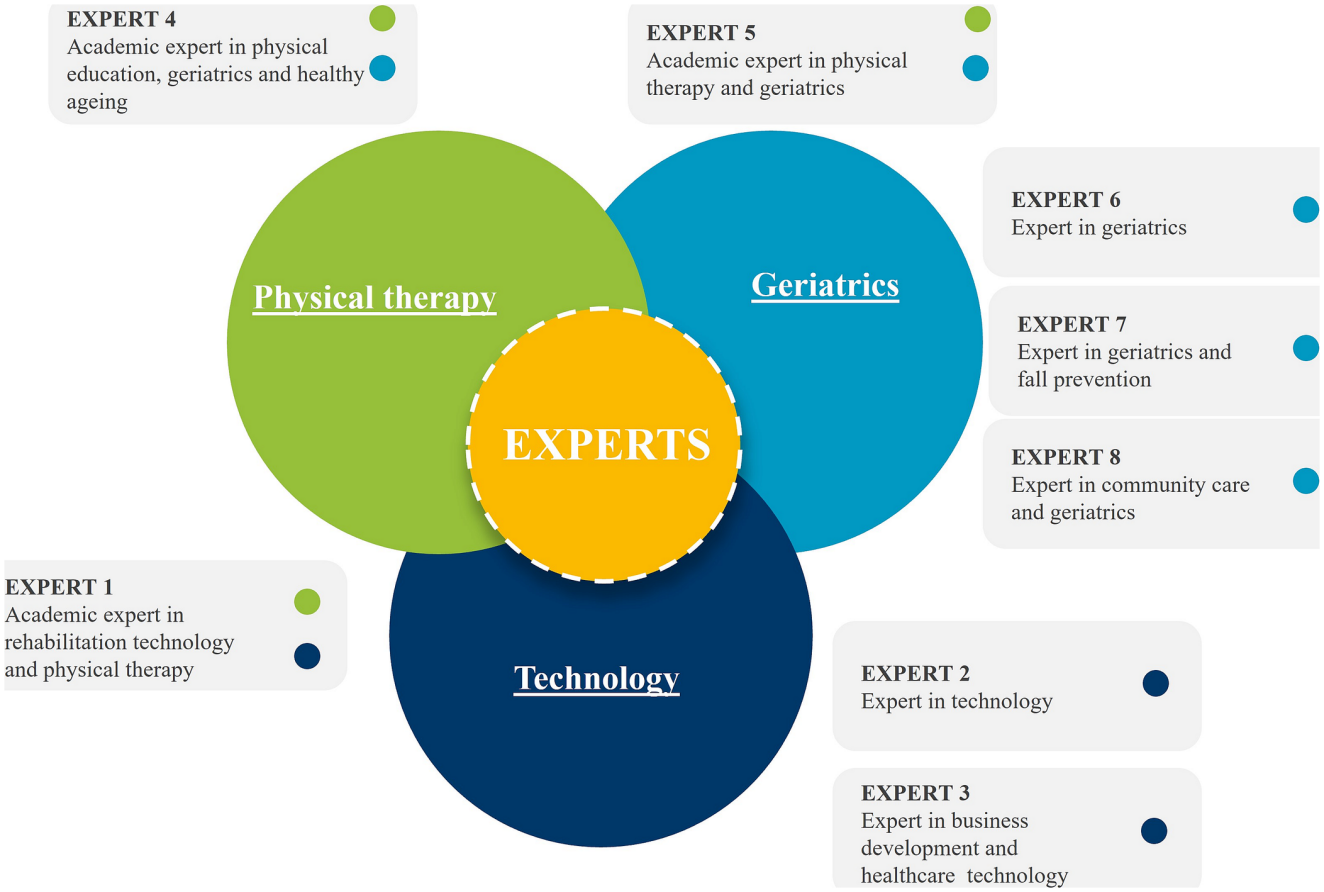
Characterization of the experts in cocreation session 2.

A co-creative process was then carried out to design a mHealth app aiming to promote PA among older adults. The study involved 16 older adults in the first session, 6 older adults plus 8 experts were then included in the second session to expand the process. The diverse range of expertise possessed by the domain experts is outlined in the Figure 2. Both sessions lasted for 4 hours each and were conducted in November and December of 2022. Participants were grouped into small teams of four to six participants to foster a creative and familiar environment (38, 39). The sessions focused on knowledge and opinion sharing rather than focusing solely on finding a solution.

The primary objective of session 1 was to elicit insights into the perceptions of older adults concerning a healthy lifestyle, including various coping strategies (such as activities, tools, and social coping). Additionally, the session aimed at identifying the key features that a mHealth app should possess to enhance PA.

Session 2 focused on further validating the findings and delving more specifically into the details of the app’s design (such as appearance, pitfalls and related strategies) (40).

As both sessions were completed the content related to the selected topics was transcribed, deductively coded and clustered by the first and second author. The deductive coding was continuously discussed among the involved researchers to ensure coherence and accuracy of the deductive coding. Finally, the clustered outcomes were organized according to the taxonomy of the Behavior Change Wheel (BCW) (41).

#### 2.2.3 Testing phase: initial prototype test

This initial prototype test was designed as an exploratory test to assess the practicality, first impression and user experience, and challenges associated with the use of the developed prototype. Participants were equipped with pre-installed app tablets to ensure device uniformity. They were given approximately 1 hour to explore the application. Participants received explicit instructions for their interaction with the mHealth app. They were first directed to open and navigate through the app. Subsequently, participants were encouraged to explore it extensively, implying a thorough examination of various sections, menus, and options. Furthermore, they were prompted to try some actions on the app, underscoring the importance of active engagement with the app’s content.

During this time, researchers observed the participants closely and employed the “think aloud” method, encouraging them to verbalize their thoughts, experiences, and impressions as they interacted with the app. This method allowed us to gain insights into their real-time experiences and thought processes while using the app. Following this explorative hour, participants were asked to provide feedback through structured surveys. A survey consisting of 22 questions, including the User Experience Questionnaire (UEQ) (42) and System Usability Scale (SUS) (43), combined with specific questions (*n* = 14) was used to evaluate users’ perception about the newly developed mHealth app. The UEQ consists of 26 items that evaluate the app across six scales: Attractiveness, Perspicuity, Efficiency, Dependability, Stimulation, and Novelty. Each item utilizes a 7-point scale, with opposing labels such as “boring” and “exciting.” The overall evaluation of each item by the participants determined the analysis outcomes.

The SUS questionnaire comprises 10 items with five response options, ranging from “Strongly agree” to “Strongly disagree.” A SUS score exceeding 68 is considered above average, while scores below 68 are deemed below average.

As this was an initial prototype test to evaluate the mHealth app in its early stage, to allow for iterations and adaptions, a convenience sample of 65 older adults was recruited with no formal sample size calculations.

## 3 Results

### 3.1 Empathy and defining phase: in-depth interviews

**Figure 3:**
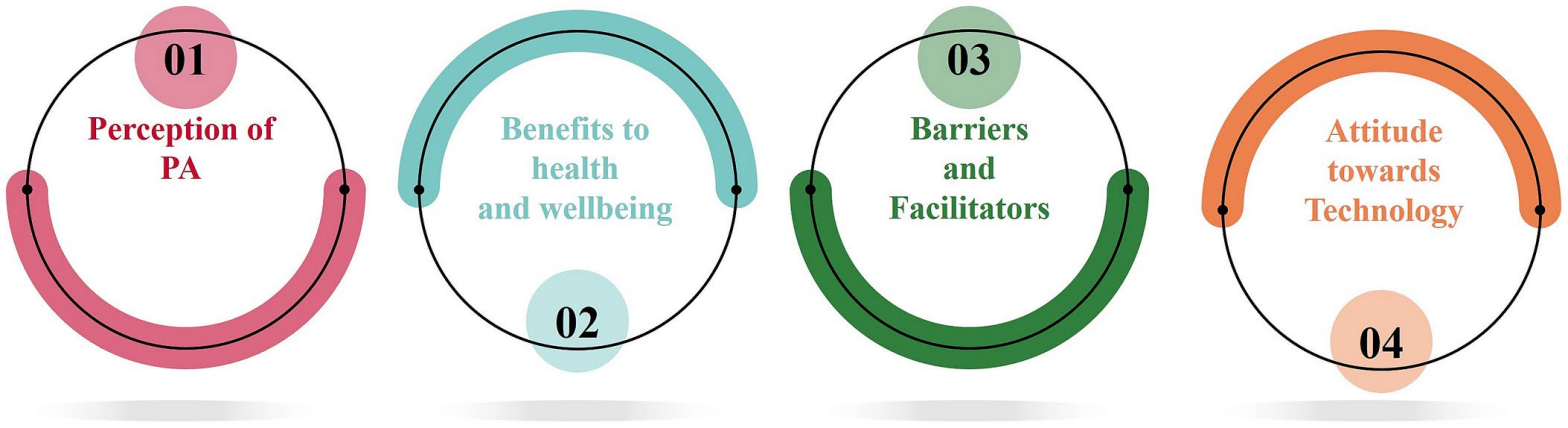
Resulting themes from the data analysis of the sensemaking phase.

Of the twenty-two older adults 37.5% reported to meet the WHO guideline for PA, while 25% were categorized as inactive (Table 1). Thematic analysis of the data resulted in the identification of four major key themes (Figure 3).

#### 3.1.1 Perception of physical activity

One of the most important findings of this part was that PA was perceived in different ways by the older people. Here are a few examples extracted from the interviews.

A woman, living with her husband in house with a big garden, said the following when asked about how she sees physical activity: *“For me physical activity entails being active around the house, doing chores, working in the garden. I think it is all the things you do from when you get up to when you go to sleep.”* Another man, who lives in an apartment, replied*: “Physical activity needs to be purposeful. For instance, walking to the bakery or going to the market.”* A widow, living in an apartment on her own, gave following response: *“Physical activity are all the sport activities I do. I go to the gym two times a week, go swimming with a friend once a week and try to walk as much during the day as I can.”* Another woman said, when asked about her physical activities and meeting the WHO guideline: *“I am very active, I clean every day. I do not see the point of doing any additional physical activities. Also, I do not have the time. I’m too busy doing other things.”*

However, notwithstanding they all agreed that being physically active is important to stay healthy.

#### 3.1.2 Benefits to health and wellbeing

In this second major theme, all participants unanimously agreed that regular PA is highly advantageous for maintaining good health. They reported various health benefits such as enhanced mobility, balance, and a reduced risk of falls. One participant shared, *“Being more physically active has improved my balance and I can get out of my chair more quickly. I’ve also noticed that I stumble less often.”* Moreover, the interviewees highlighted the positive impact on cardiovascular fitness, increased independence, and successful pain management. One participant commented on the significant improvement in his back pain since attending physiotherapy for knee arthritis, stating, “*I used to wake up every morning with pain in my back and shoulders, but now it bothers me much less.*”

Seven participants also identified the social component of PA as a very important one. One participant said: *“I go to the gym every week. It is really nice, we all know each other, we have a chat, drink a cup of coffee afterwards and it makes my day.”* Another participant mentioned: *“The nice thing is, we motivate each other.”*

Improved mental health was also cited as a significant benefit of PA, aligning with the WHO’s definition of health, which states that health is a state of complete physical, mental, and social wellbeing, and not merely the absence of a disease of infirmity (44). As one participant explained: *“When I go for a walk with a friend, there is lots of laughter, we support each other and that’s really important as you grow older and your circle becomes smaller.”* This highlights the positive impact of PA on not only physical health but also emotional well-being and social connectedness, emphasizing its contribution to overall health as defined by the WHO.

#### 3.1.3 Barriers and facilitators

Reported barriers to engage in PA included subjective poor health, lack of time, lack of perseverance, bad weather, fear of falling, and the absence of a friend to exercise with. Additionally, some participants (*n =* 6) expressed a preference for engaging in PA through enjoyable activities like walking or gardening, rather than structured exercise programs. As one participant explained, *“I really need to have a goal, just being active to be active will not motivate me. If it’s, for example, to go for a drink, I can perhaps take the bike.”* This highlights the importance of incorporating enjoyable activities into exercise routines to increase motivation and adherence. It also suggests that exercise programs should be tailored to an individual’s preferences and needs to maximize their benefits.

On the other hand, factors that enabled PA included a feeling of improved health, enjoyment of activities, better physical health, and increased social connections. These factors can act as motivators and facilitate a person’s ability to engage in regular physical activity.

#### 3.1.4 Attitude towards technology

The vast majority of participants (91%, *n =* 20) did not exhibit a high level of technology readiness when it came to using technology to assist in their PA habits. However, they were generally receptive to the idea of using an app, but simply had not considered it yet. As one participant explained, *“I only use my phone to call my daughter or play Sudoku. But I would be open to using it to move more.”* Another participant expressed interest in new technology but emphasized the importance of it being user-friendly and easy to read. Although most respondents used technology for phone calls, text messaging, and email, none of them reported using apps to track their physical activity. Some participants employed alternative mobile applications. These apps included social media platforms (*n =* 4), navigation tools (*n =* 5), communication software (*n =* 16), identity verification applications (*n =* 8), mobile banking platforms (*n =* 7) and creative tools (*n =* 3), among others. Also, participants were open to the idea of using new technologies to improve their PA habits.

### 3.2 Ideate and prototyping phase: co-creative workshops

The first co-creative session encompassed 16 older adults with a mean age of 68 ± 3 years (see Table 1 for complete information) from which 58.3% was classified as being physically active according to the WHO guideline. Moreover, a considerable majority (93.5%) of the older adults possessed a smartphone, tablet (53.3%) and/or computer (87.5%).

During the second session, a group of eight experts (see their respective expertise in Figure 2) was invited. Additionally, six older adults (mean age of 72 ± 6 years) participated. It is to note that none of them met the WHO guideline for PA. Furthermore, a large proportion of the older adults (83.3%) owned a smartphone.

**Figure 4:**
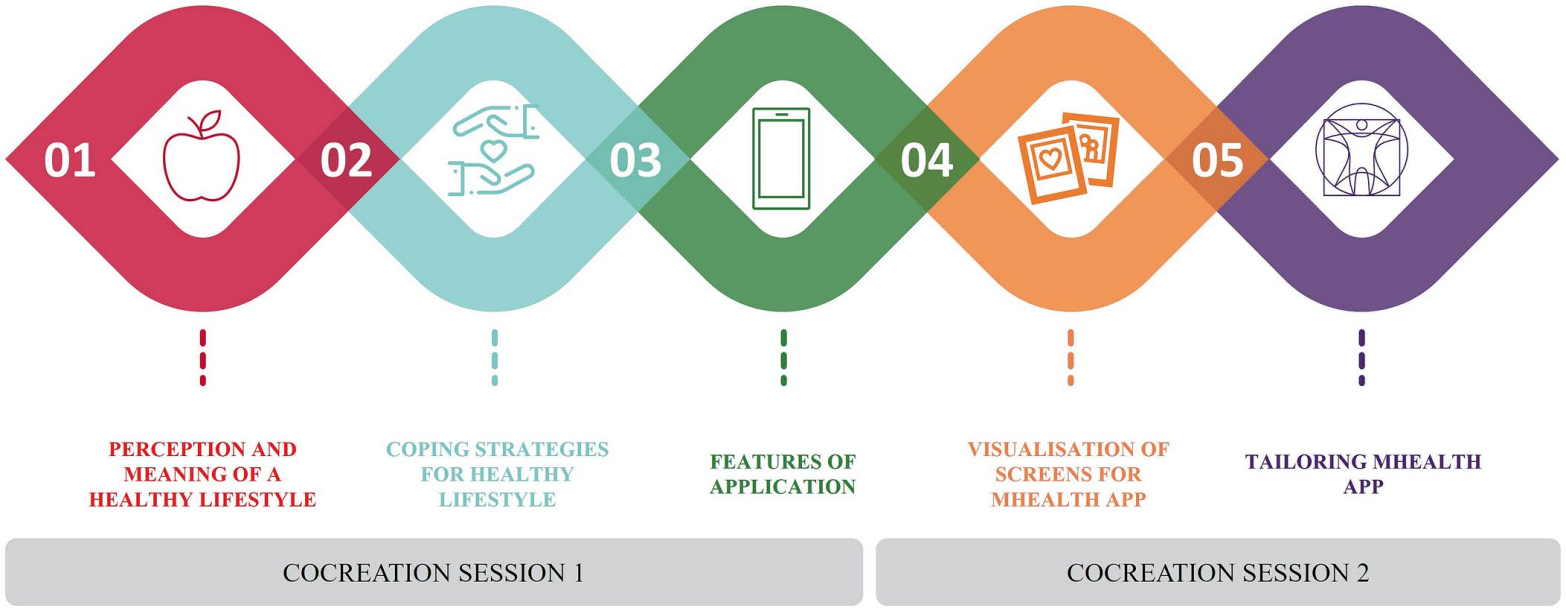
Main topics of the two co-creation sessions.

Data analysis of the workshops resulted in the identification of 5 main topics (Figure 4) that we are going to describe more into detail.

#### 3.2.1 Perception and significant of a healthy lifestyle

As individuals become older, their perception of what constitutes a healthy lifestyle can shift. This was observed in both co-creation sessions. Most participants tend to prioritize maintaining their physical and cognitive health, often through engaging in regular exercise, eating a balanced diet, and staying socially connected, enough sleep (illustrated in Table 2).

**Table 2:** Perception of a healthy lifestyle and the way older adults determine this.

Items	Description	Example
How do you stay healthy?		
Enough sleep/rest	Enough rest and sleep refers to obtaining a sufficient amount of quality sleep to support overall health and well-being	*“Living a healthy lifestyle as a senior starts with prioritizing sleep. I’ve learned that adequate rest boosts my energy levels.”* *“Sleep is the secret ingredient to my healthy lifestyle. When I get enough rest, I find it easier to make better food choices and stay committed to regular exercise. It’s like a domino effect – once I sleep well, other healthy habits fall into place.”* *“I used to underestimate the impact of sleep on my health, but as I’ve aged, I’ve realized how vital it is. When I prioritize rest, I have more mental clarity, and I can engage in social activities and hobbies that keep me active and fulfilled.”* *“As an older adult, I’ve experienced how lack of sleep can disrupt my entire day. It affects my mood, focus.”* *“In my youth, I could get away with sleepless nights, but now I know better. As a senior, I view enough sleep as an investment in my health. It’s like giving my body the time it needs to reset and prepare for the challenges and joys of each new day.”*
Healthy Diet	It includes a balanced mix of nutrients from whole foods, emphasizing fruits, vegetables, lean proteins, healthy fats, while limiting processed foods, sugars, and staying well-hydrated. It also refers to a dietary approach that aims to limit or eliminate added sugars from their daily food intake	*“As a 70-year-old, staying active and maintaining my health is a priority. For me, a healthy diet means enjoying plenty of colorful fruits and vegetables.”* *“In my 80s, I find joy in gardening and dancing. Eating well is essential for me to keep my body and mind sharp. A healthy diet, which includes lots of leafy greens and berries, helps me feel more connected to the activities I love.”* *“As an active 68-year-old, I enjoy hiking and biking. My healthy diet involves plenty of water, whole grains, and fresh products from the market.”* *“At 66, I cherish my daily walks with my dog. A healthy diet that focuses on whole foods, like fresh fruits and grilled chicken, enables me to enjoy these precious moments with my loyal friend. I also try to minimize my alcohol intake”*
No alcohol	Limited alcohol intake means moderation and being mindful of individual limits. Prioritizing hydration, understanding potential interactions with medications, and making balanced dietary choices are essential	*“For me, limited alcohol intake means enjoying a glass of wine occasionally during social gatherings, but always in moderation. It’s about savoring the moment without compromising my overall well-being.* *“I’ve always been an active person, and that has not changed in my 60s. To maintain my lifestyle, I’ve made a conscious decision to keep alcohol consumption in check. It’s not about depriving myself, but rather respecting my body and staying in control.”* *“I’ve seen the impact of excessive alcohol on friends’ health, and it made me reconsider my own habits. Moderation is the key for me now, and I see limited alcohol intake as an essential part of my active lifestyle.”* *“I’ve always enjoyed a good glass, but as I’ve grown older, I’ve become more aware of my body’s responses.”*
Regularity	Refers to maintaining consistent habits and routines in physical activity, nutrition, and overall wellness	*“For me, sticking to my daily exercise routine, whether it’s a morning walk, yoga, or even dancing is important.”* *“Being consistent with my balanced meals is key to my healthy lifestyle. I make sure to eat plenty of fruits, vegetables, and lean proteins every day. It not only nourishes my body but also helps me maintain a healthy weight.”* *“Regular check-ups and screenings are non-negotiable for me. It’s essential to catch any health issues early on, and it gives me peace of mind knowing that I’m taking care of myself proactively.”* *“Having a regular exercise buddy keeps me motivated and accountable. We support each other in staying active, making the journey to a healthy lifestyle all the more enjoyable.”*
Exercise	Activities that focus on maintaining mobility, enhancing strength and balance, and promoting overall well-being. These include aerobic activities, strength training, flexibility, balance and stability training, core strengthening, cognitive activities, and social interaction, tailored to individual abilities and health considerations	*“As I’ve aged, I’ve come to realize the importance of exercise in maintaining my independence. It’s not just about physical health; it’s about being able to take care of myself and stay self-reliant for as long as possible.”* *“Staying active has given me a new sense of community. I’ve made so many friends through group exercises, and it keeps me socially engaged and connected with others.”* *“I used to think aging meant slowing down, but exercise has proven me wrong. I’ve gained strength and flexibility, and I feel more agile now than I did a decade ago.”* *“When I look at my peers who do not exercise regularly, I can see the difference it makes. I feel like I have more energy and vitality, and it gives me the confidence to embrace aging with grace.”*
How do you determine a healthy lifestyle?		
Common Sense	Refers to the practical and intuitive understanding of making sensible choices that promote well-being and vitality in their daily lives. It involves incorporating simple, practices that contribute to maintaining and enhancing physical, mental, and emotional health	*“I’ve learned to use common sense by listening to my body. If I feel tired, I rest, and if I feel hungry, I eat.”* *“Common sense helps me make sensible food choices. I focus on eating a* var*iety of colorful fruits and vegetables and avoid processed foods as much as possible. It’s about keeping it simple and sticking to foods that I know are good for me.”* *“Using common sense means knowing my limits. I do not push myself too hard, but I also do not let age be an excuse to be sedentary. I find a balance between staying active and giving myself enough rest.”* *“I’ve always believed in the power of common sense when it comes to health. It’s about moderation in everything, from the food I eat to the time I spend on screens. Finding a healthy balance makes a difference in how I feel.”*
Doctor	Regular visits to the doctor play a significant role in helping older adults stay on track with their health goals and make informed decisions about their well-being as they age	*“I make it a point to visit my doctor regularly to discuss my health goals and get personalized advice.”* *“I trust my doctor to give me the best advice. They help me navigate through the overwhelming amount of information out.”* *“Whenever I have concerns about my mobility or managing pain, I turn to my doctor for help. They offer suggestions on exercises and treatments that alleviate discomfort and improve my overall mobility.”* *“My doctor is my go-to resource for staying up-to-date with the latest health information. They separate fact from fiction, so I can make informed decisions about my lifestyle choices.”*
Intuition	It is a valuable and instinctive tool in determining an active healthy lifestyle. Intuition, in this context, refers to their inner sense of knowing and understanding what feels right for their bodies and overall well-being	*“My intuition helps me set personal boundaries for my health. I know when to say no to commitments that might drain my energy or affect my well-being, allowing me to prioritize self-care.”* *“I’ve noticed that my intuition often leads me to seek professional advice when I sense something might be off with my health. Trusting my instincts has helped catch health issues early and receive timely treatment.”* *“When it comes to staying socially active, I listen to my intuition to nurture relationships with people who uplift and support me. I surround myself with positive influences, which positively impacts my mental health.”* *“I’ve found that my intuition often aligns with my body’s needs. It reminds me to prioritize rest and listen to my energy levels, allowing me to maintain a balanced and sustainable lifestyle.”*
Articles/newspaper	Articles and newspapers are seen as valuable sources of information and inspiration in relation to determining an active healthy lifestyle by older adults. They turn to these written materials to stay informed about the latest trends, scientific research, and expert advice related to health and wellness for seniors	*“I make it a habit to read health articles and watch the news to stay up-to-date with the latest health trends and scientific discoveries. It helps me make informed decisions about my diet, exercise, and overall well-being.”* *“Reading about nutrition in newspapers helps me make healthier choices when grocery shopping and preparing meals. It’s a practical way to ensure I’m eating foods that benefit my body and mind.”* *“Watching health-related segments on the news keeps me informed about the latest breakthroughs and medical advancements. I feel more confident discussing health matters with my doctor armed with this knowledge.”* *“Newspapers often have local events and programs for seniors. I use this information to join fitness classes and workshops, fostering a sense of community and staying motivated to stay active.”*
Being able to do regular exercise	Being able to exercise is a critical factor that older adults use to determine their overall health and well-being. For many seniors, the ability to engage in regular physical activity is a strong indicator of their health status and functional capacity	*“I know I’m healthy when I can keep up with my daily walks. Being able to exercise without too much strain makes me feel vibrant and confident in my overall health.”* *“For me, being able to exercise means I have the energy to enjoy time with my grandkids and take them on outdoor adventures. It reassures me that I’m in good health and can actively be a part of their lives.”* *“I gauge my health by how easily I can move and stay active. When I can do my gardening and take care of household chores without feeling exhausted, I know my body is in good shape.”* *“I use my ability to exercise as an indicator for my health because it enables me to stay independent and do the things I love. As long as I can stay active, I know I’m taking care of myself.”*

One participant in the study, a 75-year-old woman, stated that *“it’s important to eat well and exercise regularly so I can stay active and independent. “*Similarly, a 68-year-old man commented that “*staying active and socializing with friends and family keeps me feeling young and healthy*.” Older adults also recognize the importance of mental and emotional health, with one 80-year-old woman saying, *“keeping my mind sharp through puzzles and reading helps me feel happy and fulfilled.”*

Overall, participants perceived a healthy lifestyle as one that prioritizes physical and cognitive health, as well as social connections and emotional well-being. They stated that these factors contribute to their overall quality of life and ability to maintain independence as they age.

#### 3.2.2 Coping strategies for a (more) healthy lifestyle

In session 1, participants brainstormed about how they stay active and healthy and which activities they engaged in.

Depending on the challenges that arise with age, the session revealed that older adults employ a variety of coping strategies to stay healthy and active. Some participants emphasized on the importance of PA, stating *“I try to do some form of physical activity every day, whether it’s walking, gardening, or stretching. It keeps me feeling good and helps me maintain my independence.”* Social support was also found to be a crucial factor, as another participant shared*, “I love going to the service center and participating in group exercise classes. It’s fun and helps me stay accountable to my fitness goals.”* It became clear that family, friends and community support plays an important role in helping older adults stay motivated and engaged.

Finally, technology was found to be a useful tool for maintaining healthy habits by some of the participants, as one participant explained, *“I love using my Fitbit to track my steps and see how much activity I’m getting each day. It helps me stay accountable and motivated to stay active.”* These findings highlight the importance of a multifaceted approach to promoting PA and healthy habits among older adults.

#### 3.2.3 Features of the mHealth app

A brainstorming exercise, *“What’s on your radar”* (Table 3), was performed to explore the potential future design and content (i.e., format, content, support, social aspect and other unlisted topics) of the mHealth application.

**Table 3:** What’s on your radar brainstorm.

Format	Content	Support	Social Aspect	Joker*
Move TV	Exercise video’s	Correct and clear explanation	Community	Anytime anywhere
You Tube	Event calendar	Clear video	Contact with other users	Affordable / for free
Laptop or tablet	Program of 10 min, not too long	Doctor promoting app	Phone call from a friend	Personal coach
Easy in use	Achievable exercises	Push notifications	Peer had to perform exercise	Healthy easy recipes for cooking
Readable, large letters	Target oriented	Tailored motivation	Chat platform	Daily activities
Structured exercise	Giving feedback and motivation	Visible results	Guidance from physical therapist	Combining with mind games
User-friendly exercises	Safe	Feedback	Video call with coach	Pleasure reward
Instruction video	Evidence based	Reimbursement	Chatbot as a virtual coach	Simple
	Informative on why to exercise	Digital coach		Easy instructions

*The term “joker” was used by participants in this study to describe something that did not fit into existing categories, going beyond the usual boundaries of classification.

##### 3.2.3.1 Format

Participants preferred a format which is easy to use, visually appealing, easy to read, and accessible on a variety of devices. One participant explained “*I want it to be accessible on my smartphone and tablet, so I can use it wherever I go.”*

##### 3.2.3.2 Content

Participants emphasized the importance of content that is engaging, easy to understand, and personalized to their needs. One participant noted, “*I want the app to give me exercises that are tailored to my physical abilities and health conditions. It has to be safe. I also want it to be fun and interactive, so I stay motivated to use it.”*

Another participant stated*: “I would like an app that recommends exercises that are tailored to my physical abilities and that change as I get stronger*.” They also preferred personalized exercise plans and instructional video**s** to ensure proper form and technique. One participant said, *“It would be nice to have exercises that you can do while doing other things. For instance, like bending your knees ten times when brushing your teeth.”*

Participants were asked to rate (on a scale of 1 to 5) the importance of various content aspects. The ranking showed that exercises were considered the most important aspect by all participants, followed by information, design, technical features, and a reward system.

##### 3.2.3.3 Support

Participants identified the importance of ongoing support, with features such as (daily) reminders and progress tracking. One participant explained, *“I want the app to send me reminders to exercise and track my progress over time. It would help me stay on track and motivated to continue exercising.”* Another participant said: *“I want the app to compose the exercises itself. Also, support from a coach or physiotherapist would come in handy, as I do not know anything about exercise myself. I’d rather rely on experts.”* A third person stated,” *It would be nice to get kudos when I have done all my training sessions.”*

##### 3.2.3.4 Social aspect

Social support was identified as a key feature, with participants emphasizing the importance of connecting with others who share similar health goals in a collaborative rather than competitive environment. One participant noted, “*I want the app to have a social aspect where I can connect with other older adults who are trying to stay active. It would be great to have a community where we can support each other and share tips and encouragement.”* Several participants also mentioned that it would be useful if the app had a calendar with all events in the area, as now they have to keep searching on different websites.

#### 3.2.4 Design of the mHealth app

To explore the visualization and screens for the mHealth app, mock ups were used to illustrate different potential designs.

Participants preferred the mHealth app to be easy-to-use and navigate, with clear fonts and large buttons. One participant stated, *“I want an app that I can easily understand and use without having to ask my daughter ten times how it works.”* Another participant stated **“***I want the app to have big buttons and clear fonts, so I do not have to strain my eyes*.” An expert stated, “*Choosing the right color schemes with enough contrast is critical for older adults, who may have difficulty distinguishing between certain colors.”*

Another older participant emphasized the importance of ease-to-use, stating, *“I do not want the app to be too complicated or overwhelming. I want it to be user-friendly and easy to navigate.”*

Finally, it became clear that daily reminders to exercise and progress tracking to monitor progress over time also should be incorporated and should be easy and quick to read.

#### 3.2.5 Tailoring motivational notifications for the mHealth app

Participants emphasized the importance of notifications being tailored to their specific needs and preferences. The content, delivery method, and wording of these messages were deemed crucial, with one participant stating, “*I do not want to be bombarded with notifications that are irrelevant to me*.” Another participant emphasized the importance of the timing of the notifications, stating, “*I want notifications to be sent at a time that is convenient for me, so I can engage in PA without disrupting my daily routine.”*

Additionally, participants highlighted the importance of notifications being motivational and encouraging, with one participant stating, “*I want the app to send positive messages that motivate me to engage in physical activity.*” Another participant emphasized the importance of notifications being supportive, stating, “*I want the app to send messages that make me feel supported and encourage me to keep going.”*

Some participants expressed discomfort with pushing-messages such as “You need to exercise.” They found such messages intrusive and disliked the idea of a stranger dictating what they should or should not do. Some participants stated that they would appreciate receiving messages from healthcare professionals or friends.

Participants also expressed the importance of notifications being easy to understand and access. One participant stated, *“The notifications should be easy to understand and not too technical, so I know exactly what I need to do.”* Another participant stated “*I want the app to send notifications that I can access easily and quickly* (e.g., via *e-mail*)*, so I do not have to spend too much time looking for them*.

### 3.3 The mHealth app, MIA, more in action

**Figure 5:**
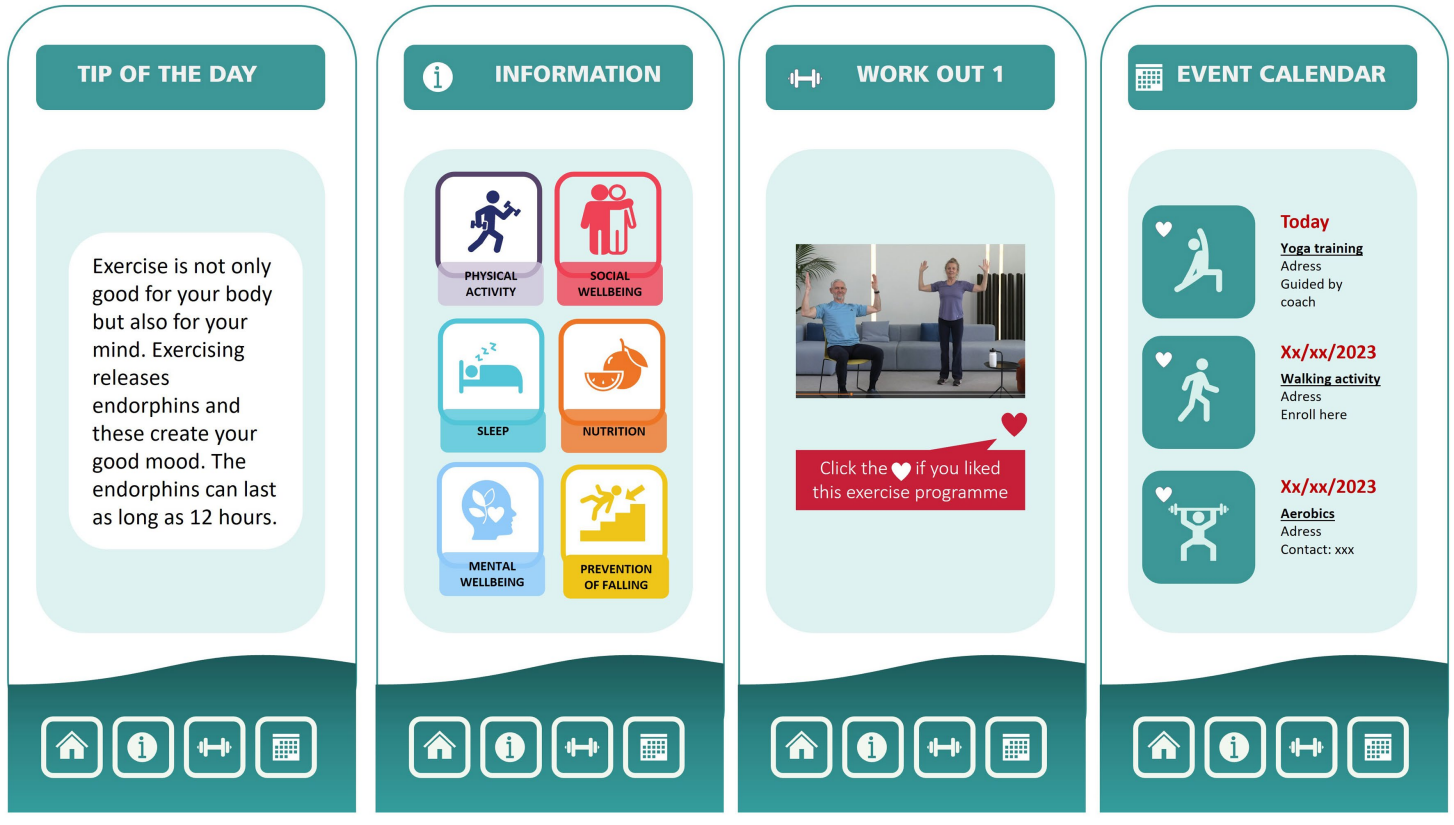
Visualization of the mHealth app on promoting physical activity and an active healthy lifestyle in older adults.

Through a collaborative co-creation process, a mHealth app was developed and refined over multiple iterations with the goal of promoting physical activity and fostering a lifestyle centered around an active and healthy approach. Content and design were derived from both the co-creative workshops and based on the theoretical framework of the intervention functions of the BCW. The final mHealth app is composed by four major features: tailor-made tips, literacy aiming to improve awareness about PA and an active healthy lifestyle, exercise workouts and a community calendar aiming to enhance social connections. To customize the user experience, a brief personal description was created as the start screen, which prompts older adults to answer questions related to their baseline physical activity level, motivation, and medical history. Figure 5 visualizes the different features of the mHealth app.

#### 3.3.1 Tailor-made motivational tips

To support the target population, the mHealth app includes a goal-setting function that allows users to set and track progress towards individualized physical activity goals. In addition, the mHealth app provides daily reminders, tailored feedback and encouragement through push notifications, which are delivered at times that are most likely to be effective in promoting behavior change. To avoid the risk of repetition and maintain engagement, the mHealth app incorporates a database of over 365 messages, each tailored to the user’s individual needs and preferences. In order to support the users autonomy and competence, such as stated by the BCW (45), they were able to choose their own goals and work-outs videos. This flexibility made it possible for the user to adapt their weekly physical activity plan to fit other activities in their life and feel in control of the activity.

#### 3.3.2 Raising awareness and providing information

The co-creative process revealed that PA was perceived in different ways by the older adults. It is of note that many of them were also not aware of the benefits of PA and lacked knowledge of how to incorporate PA into their daily routine. Therefore, a specific feature was included in the mHealth app to inform older adults about the benefits of PA on physical, mental and cognitive wellbeing (health literacy). By offering information to improve awareness about PA and translating scientific studies into a language that older adults can easily understand, the latest scientific findings on the benefits of PA can be communicated. By providing accurate and easily understandable information older adults are facilitated in making informed decisions about their health, leading to a better quality of life.

#### 3.3.3 Exercise work-outs

Aiming at promoting PA in older adults the feature ‘Work-Out videos’ was a crucial part of the mHealth app. The videos were recorded in a home-like recognizable environment, as this was deemed to be more familiar and comfortable for the target population. They were carefully designed to feature peers who demonstrated the exercises at three different levels (beginner, medium, and expert) to accommodate a range of physical abilities. Verbal instructions were also provided to complement the video demonstrations.

#### 3.3.4 Community calendar

The importance of social interaction in the context of PA among the population of older adults was highlighted during the co-creative process. Exercising with peers was seen as a facilitator of being more physically active. Therefore, in order to foster social cohesion and encourage PA, a community calendar feature was developed for the mHealth app. This feature enables sports organizations, civic communities, cities and other initiatives to share information about sports activities taking place in the local neighborhood. Thereby providing users with a comprehensive overview of the activities that may be of interest to them during the week and promoting accessibility and interest among the older adults.

### 3.4 Testing phase: initial prototype test

To gather valuable insight, a total of 65 older adults were invited to participate, test and evaluate the prototype of the mHealth app. Demographic characteristics of the participants are provided in Table 1.

According to the System Usability Scale (SUS), the initial version of the mHealth app was considered “acceptably good” with a mean score of 70.38 ± 14.51. The assessment of the individual features of the mHealth app, as presented in Table 4, unveiled encouraging feedback from participants regarding its user-friendliness, seamless integration of functions, and ease of learning the mHealth app.

**Table 4:** Means and standard deviation for the system usability scale.

Questions	SCORE Mean ± SD
I think I would like to use the app frequently	3.52 ± 1.06
I found the system to be unnecessarily complex	1.78 ± 1.12
I thought the system was easy to use	3.89 ± 1.10
I think that I would need support of a technical person to be able to use the system	2.11 ± 1.30
I found the various functions in the system were well integrated	3.55 ± 1.01
I thought there was too much inconsistency in the system	2.03 ± 0.97
I would imagine that most people would learn to use the system very quickly	3.52 ± 1.07
I found the system very cumbersome to use	1.961 ± 1.18
I felt very confident using the system	3.31 ± 1.12
I needed to learn a lot of things before I could get going with the system	1.75 ± 1.13
System usability scale total score (out of 100)	70.38 ± 14.51

* Responses were scored in a 5 – point Likert Scale: 1 = strongly disagree – 4 = agree, 5 = strongly agree.

**Figure 6:**
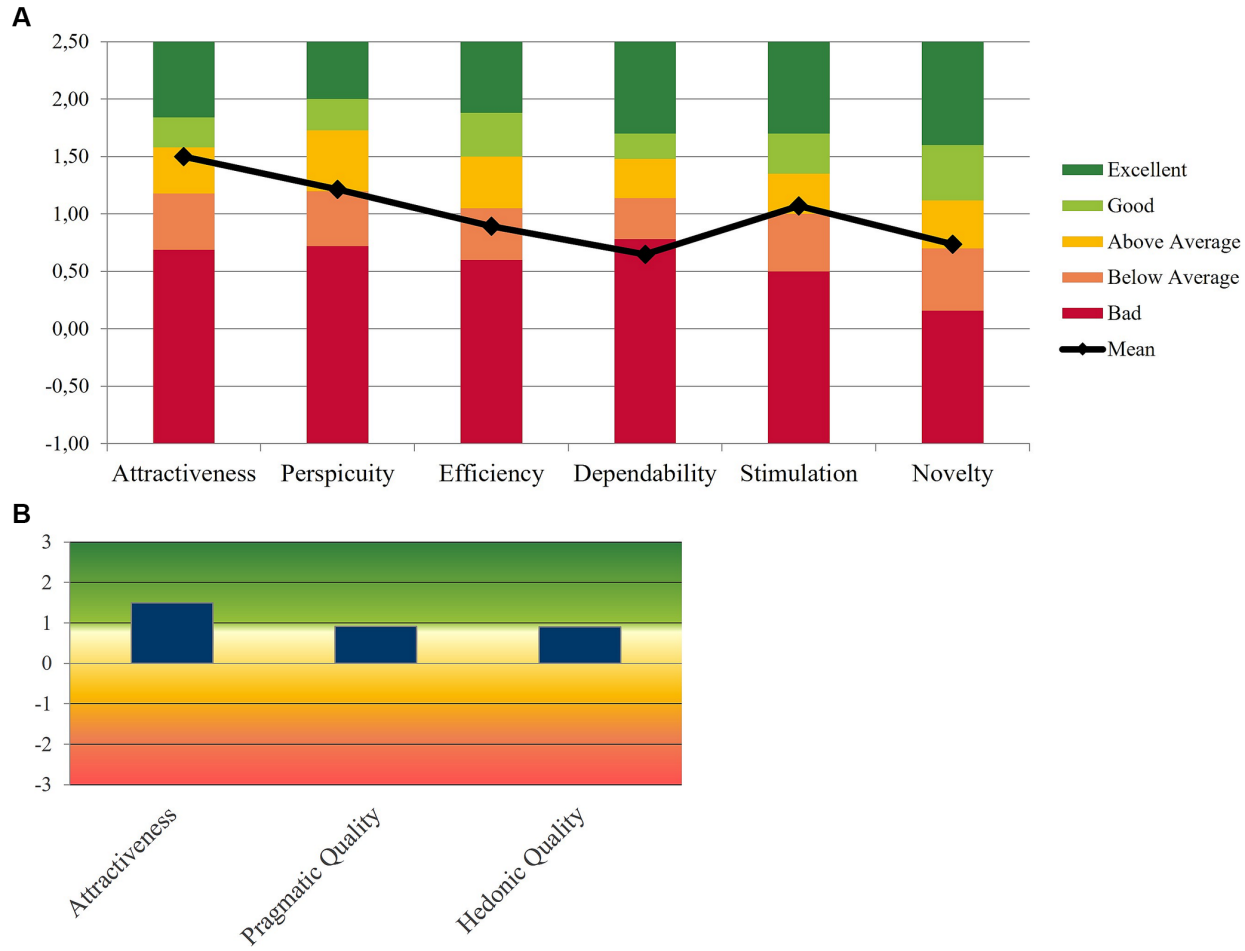
Results of the UEQ scale. **(A)** The value of each UEQ item for the whole sample compared to the UEQ benchmark dataset, **(B)** the overall UEQ results by grouping the UEQ scales into 3 categories: Pragmatic quality; Hedonic quality; Attractiveness.

The complete results of the User Experience Questionnaire (UEQ) are presented in Table 5. To assess the mHealth app’s performance in relation to other products (46), Figure 6A displays the relative results of the UEQ compared to the UEQ benchmark dataset.

**Table 5:** The prototype app’s use metric evaluation.

	Total (*n* = 65)
	Mean ± SD
SUS score	70.38 ± 14.51
UEQ score	
Attractiveness	1.50 ± 1.14
Perspicuity	1.21 ± 1.09
Efficiency	0.89 ± 0.78
Dependability	0.644 ± 0.80
Stimulation	1.07 ± 1.51
Novelty	0.74 ± 1.13

Across all evaluated aspects, attractiveness received the highest score with a median score of 1.7 [Inter Quartile Range (IQR) = 1.29]. The mHealth app demonstrated an overall average score of 1.2 [IQR = 1.09] for perspicuity, 0.90 [IQR = 0.78] for efficiency, 1.25 [IQR = 0.75] for dependability, 1.75 [IQR = 1.12] for stimulation and 1.69 [IQR = 0.5] for novelty. Figure 6B further illustrates the overall UEQ outcomes by categorizing the scales into three distinct categories: Attractiveness, representing the mHealth app’s appeal, pragmatic quality (perspicuity, efficiency, and dependability), which captures the mHealth app’s functional aspects, and hedonic quality (stimulation and originality), encompassing the app’s non-task-related qualities.

More general statements were assessed using a Likert scale ranging from 1 (strongly disagree) to 5 (strongly agree) in relation to four features: tailor-made tips, exercise workouts, raising awareness and providing information, and the community calendar, along with their respective screens. The overall scores are presented in Table 6, revealing that 7 out of 11 statements scored above 4 on average. The exercise workouts and the community calendar were the top-rated features among participants. The workout experience received positive feedback, with an average score of 4.27 ± 0.88, stating that the instructions provided were clear (4.19 ± 0.84) and the difficulty level of the exercises appropriate (4.10 ± 0.91). Furthermore, participants expressed keen interest in the community calendar, finding it visually appealing (4.13 ± 0.94). They emphasized the importance of staying informed about exercise and sport activities their neighborhood (4.15 ± 1.05) and believed that having a clear overview of activities would motivate them to participate more frequently (4.16 ± 0.87).

**Table 6:** Participants ‘perceptions of the prototype of the mHealth app.

Statements	Scores Mean ± SD
Tailor-made motivational tips*	
The “tip of the day” is clearly readable to me	3.97 ± 1.13
I am going to follow up on the tip of the day	3.38 ± 1.12
Exercise work-outs*	
The explanation of the workout was clear so you could join them smoothly	4.19 ± 0.84
The difficulty level of the exercises (how smoothly you could perform them) was good	4.10 ± 0.91
I enjoyed doing the workout	4.27 ± 0.88
Raising awareness and providing information*	
The categories, exercise, social wellbeing, sleep, nutrition, mental wellbeing and fall prevention are complete	3.89 ± 0.96
I find this information interesting	4.14 ± 0.84
It worked well to return to the app after viewing the information (which opened in a new screen)	3.93 ± 0.94
Community calendar*	
I would like to be kept informed about exercise and sports activities in my neighborhood	4.15 ± 1.05
I find it interesting to look at this calendar	4.13 ± 0.94
If I had a clear overview of activities in my neighborhood, I would go to such activities more often	4.16 ± 0.87

*Responses scored on a 5-point scale: 1 = strongly disagree, 5 = strongly agree.

Regarding the “raising awareness and information” feature, participants found the presented information interesting (4.14 ± 0.84). They appreciated the comprehensive range of topics, including exercise, social wellbeing, sleep, nutrition, mental wellbeing, and fall prevention covered a comprehensive range of topics (3.89 ± 0.96).

Finally, participants were asked about their likelihood of recommending the mHealth app to a friend, with a rating scale ranging from 1 to 10. The analysis revealed an average score of 7.66 ± 1.79, indicating a generally positive inclination among participants to endorse the mHealth app to others. These findings align with the fact that 72% of users reported finding the app easy to use.

## 4 Discussion

### 4.1 General consideration

The design thinking method has emerged as powerful tool in technology development (28), particularly in the field of healthcare. As the healthcare landscape evolves, it is essential to prioritize the needs and perspectives of end-users, particularly the population older adults who often face unique challenges in adopting technology. Against this backdrop, this study aimed to develop a mHealth app promoting PA and a healthy active lifestyle with community-dwelling older adults and experts guided by the design thinking method.

The increase of mHealth apps aiming to promote healthy behavior has been exponential. Nonetheless, the majority of these apps and their development lack evidence-based behavior change theories (47, 48). To enhance the effectiveness of mHealth apps, there is a growing consensus that they should be based on empirical evidence and behavior change theories, which incorporate strategies such as goal setting, barrier identification, self-monitoring, and action planning (49). These techniques have been shown to be effective in initiating behavior change among users (50). For the design thinking process of this study, the widely-used and effective theoretical framework, the BCW, was selected to guide the interventions and development of the mHealth app (41, 45, 51). The BCW helped identifying key determinants of PA behavior in older adults and enabling the selection of appropriate intervention strategies, resulting into four features for the mHealth app, which included tailored tips, raising awareness and providing information, exercise workouts with peers and a community calendar aimed at enhancing social connections. Additionally, the BCW ensured that the app development process was grounded in a clear understanding of the targeted behaviors as well as the barriers and facilitators to those behaviors.

In addition, previous research indicates that behavioral change interventions can successfully motivate older adults to engage in PA (47, 50). Nevertheless, it is important to combine these behavioral change interventions with meaningful motivators, including social and environmental support, as well as the personal satisfaction derived from physical activities. To attain successful outcomes in motivating older adults to adopt an active lifestyle, a comprehensive approach considering their social, individual, and environmental factors that are specific to this population is crucial and necessary (40).

### 4.2 Main results

During the design thinking process, valuable insights into several key areas were gained. These included the perception of PA among older adults and experts, coping strategies for being physically active, barriers and enablers to engaging in PA, and attitudes and expectations regarding a mHealth application’s ability to promote PA.

#### 4.2.1 Perception of physical activity

It has been previously highlighted that many older adults tend to overestimate their PA levels, which can lead to the development of interventions that may not accurately address their needs (10). While everyday activities, such as house chores and gardening can be moderately to vigorously intense, this is not always the case. Throughout the in-depth interviews, participants were asked about their level of PA and whether they perceived it to be adequate. This theme was further explored by asking participants to describe specific activities in which they were engaging. The findings indicated that, despite self-reported levels of PA, their actual activity levels did not consistently correspond with these reports. This observation made it clear that engaging in PA at the recommended intensity necessitates a correct comprehension of moderate to vigorous PA. Unfortunately, current definitions and descriptions of moderate to vigorous intensity exercise are unclear, making it challenging particularly for older adults (52). This discrepancy highlights potential limitations of relying solely on self-reported data to assess PA levels among older adults. Self-report tools may be limited by scope and subjectivity (53). Objective measures, such as wearable technology, are essential for accurately quantifying PA and addressing issues of physical inactivity in this population (54). In addition, older adults perceive PA as embedded in every day activities (41). Moreover, some older people still believe that PA is unnecessary or even potentially harmful. Others recognize the benefits of PA but report a range of barriers to PA participation. These factors must be considered when developing the mHealth app.

#### 4.2.2 Coping strategies

Engaging older adults in PA programs can be challenging and it can be even harder to maintain that commitment. The mHealth app should integrate PA into daily routines, emphasizing personal choice and activity preference, rather than intense analytical exercise routines (42). To effectively motivate older adults, the mHealth app should go beyond the physical benefits of exercise. Tailored push notifications, personalized feedback (i.e., activity trackers, digital phenotyping) and information about the health-promoting effects of PA are indispensable. Additionally, community-based activities to encourage socialization and a sense of belonging among users should be included.

#### 4.2.3 Barriers and facilitators

Older adults often face unique challenges when it comes to engaging in regular PA. One of the key barriers to PA were physical limitations (43, 44). Many older adults have chronic conditions such as osteoarthritis, cardiovascular disease or respiratory problems that can cause pain or discomfort, making it difficult for them to engage in PA. These limitations can discourage them from engaging in PA, while it is a key factor in maintaining optimal function and quality of life despite these underlying conditions. A mHealth app for promoting PA for older adults should anticipate this particular barrier.

Another barrier is the fear of injury, especially for those who have already experienced falls or injuries (45). This fear can make them hesitant to participate in PA and restrict their opportunities for exercise. This emphasizes the crucial role of literacy aiming to improve awareness about PA and an active healthy lifestyle, thus integrating accurate information, offering safe exercise options, and delivering personalized feedback within the mHealth app.

Lack of social support (e.g., engaged friends or family members) is also a barrier in engaging in PA (46–48). Without engaged friends or family members, they may feel isolated and less motivated to be physically active. Conversely, social support can be a facilitator, with family members, friends, caregivers, or joining group exercise classes or walking groups providing encouragement, companionship, and motivation (49).

#### 4.2.4 Challenges regarding the implementation of a mHealth application to promote PA

The implementation of technological solutions presents practical challenges for users who have limited experience, particularly older adults (50). The “digital divide” resulting from socioeconomic status, age, geographic location, and cultural factors must thus be taken into account (51).

Furthermore, it is essential to provide activity interventions that align with the lifestyle and expectations of older adults and offer tailored interventions based on individual preferences and capabilities. A review of older adults’ perspectives on technology revealed that personalized solutions are necessary since one-size-fits-all approaches are inadequate (52).

Intrinsic factors (i.e., control, independence, and safety requirements) as well as extrinsic factors (i.e., usability, feedback, and costs) influence the motivation to use technology. To encourage long-term technology use, the positive benefits (i.e., how the technology promotes independence) must be emphasized, and the technology needs to be perceived as reliable and effective. Given the considerable learning curve involved, it is crucial to provide adequate support to assist older adults in becoming familiar with the technology.

### 4.3 Limitations and strengths

The findings of this study have to be seen in light of some limitations First, it is important to note that the participants may not adequately represent the entire older adult population. Notably, during the initial co-creation session, there was a significant overrepresentation if women, account for 91% of the participants. For the second co-creation session none of the older adults met the minimal level of PA defined by the WHO. Overall, most of the participants have a smartphone and are using apps so they are already well familiarized and aware of the mobile technology. In the future it would be interesting to evaluate the perception of older adults less familiar with the use of new technology to determine if they are willing to adopt this mHealth app. Notwithstanding adding relevant input and ideas regarding a mHealth app, the participants who volunteered to participate may have been more motivated and interested in PA compared to the general population, thereby limiting the generalizability of the findings.

Secondly, the input of older adults may not always align with evidence-based practice, which may limit the effectiveness of the future intervention. Additionally, cocreation with older adults can sometimes result in conflicting opinions regarding the design and features of the app. In the ideation and prototyping phase (the co-creation sessions), it is important to note that the older participants exhibited a low level of PA. This aspect could potentially bias into the analysis regarding their perception of the importance of PA. Fortunately, during this phase, the opinions of the seniors were integrated with those of the experts, thus greatly reducing this potential bias.

Thirdly, it is crucial to acknowledge that the initial prototype test intervention was implemented as a one-time exploratory initiative, limiting the extent to which the results can be generalized. Due to the absence of repeated measurements or long-term follow-up and limited adherence, the findings should be interpreted with caution and further investigation is warranted to ascertain the robustness of the observed outcomes. This test was mainly conducted to facilitate the iterative process of the development of the app.

Furthermore, it is important to highlight that the lack of blinding among the assessors could introduce potential biases and influence the generalizability of the prototype test. Without blinding, there is a possibility that the assessors’ knowledge or expectations of the intervention may have influenced their assessment and subsequent interpretations of the results.

To enhance the reliability and validity of future studies, it is recommended to incorporate randomized controlled designs with blinding procedures to minimize bias and increase the generalizability of the results. Moreover, conducting longitudinal assessments or employing a multi-site approach would provide a more comprehensive understanding of the intervention’s effects over time. Finally, unrealistic expectations of the mHealth app may also be a potential pitfall. Participants or users may hold high hopes for immediate and dramatic transformations, overlooking the fact that behavior change often occurs gradually. It is important to emphasize the mHealth app’s intended role as a supportive tool in the process of change.

To mitigate this potential pitfall, proactive management of expectations becomes vital. Clear communication and educational efforts can play a pivotal role in fostering a realistic understanding of the app’s capabilities and limitations. By addressing these expectations and providing users with accurate information, sustained engagement can be promoted and the app’s effectiveness as a tool for positive change can be maximized.

Despite these limitations, a strength of this study was the inclusion of older adults themselves in developing a mHealth application aiming at promoting PA. This was yet acknowledged as being important in previous research (55). A well-structured design thinking process of co-creation with end-users can ensure the design of attractive technologies and is more efficient (56). Another strength of this study was the sense of ownership. By involving older adults in the design thinking process, they feel a sense of ownership and investment in the development of the app. This sense of ownership can increase the relevance and acceptance of the intervention among older adults (57, 58).

Lastly, the research methodology employed in this study was a combination of in-depth interviews and co-creative workshops, a prototyping and an initial prototype testing. By leveraging these techniques, rich and diverse data on participants’ experiences related to technology and PA could be collected. The qualitative collaborative approach was particularly impactful, as it offered participants a unique opportunity to engage in meaningful discussions and share insights on how they would tailor a mHealth app to their individual needs, desires and preferences. Overall, the combination of these research methods allowed us to collect comprehensive and nuanced data, which holds significant implications for the advancement of the technology-based intervention in the domain of promoting PA promotion in older adults.

## 5 Conclusion

The study yielded valuable insights in several areas. Firstly, it emphasized the importance of older adults having a correct understanding of the definition of moderate to vigorous PA, as they tend to overestimate their activity levels.

Secondly, it highlighted the importance of mHealth apps integrating PA into daily routines, focusing on personal choice and providing tailored push notifications, feedback, and information on health benefits of PA. Community-based activities that promote socialization and a sense of belonging were also found to be crucial motivators.

Thirdly, physical limitations, fear of injury, and lack of social support were identified as significant barriers, while social support, companionship, and safe exercise options were found to be facilitators. These factors should be considered when designing mHealth apps for promoting PA among older adults.

Overcoming implementation challenges, addressing, digital divide, and personalizing solutions are crucial.

In conclusion, this study demonstrates the potential of design thinking and emphasizes the importance of considering the perspectives of older adults in the development of technology-based interventions for promoting PA. By addressing the challenges and incorporating the facilitators identified, mHealth apps can play a vital role in motivating and supporting older adults in leading active and healthy lifestyles. Future research should further explore the effectiveness and long-term impact of such mHealth interventions, employing randomized controlled designs with blinding procedures and considering the holistic factors that influence behavior change in older adults.

## Data availability statement

The raw data supporting the conclusions of this article will be made available by the authors, without undue reservation.

## Ethics statement

The studies involving humans were approved by Comité voor Medische Ethiek Faculty of Medicine and Life Sciences, Hasselt University. The studies were conducted in accordance with the local legislation and institutional requirements. The participants provided their written informed consent to participate in this study.

## Author contributions

KD: Writing – original draft, Writing – review & editing, Conceptualization, Data curation, Formal analysis, Funding acquisition, Investigation, Resources, Visualization. RL: Writing – review & editing, Investigation. EK: Writing – review & editing, Investigation. NM: Writing – review & editing, Investigation. SV: Writing – review & editing, Investigation. JaB: Writing – review & editing, Software. JoB: Writing – review & editing. AS: Writing – review & editing. DH: Writing – review & editing. BB: Writing – review & editing, Methodology, Supervision, Writing – original draft.
