# Supplementary material for: Promoting physical activity and a healthy active lifestyle in community-dwelling older adults: a design thinking approach for the development of a mobile health application
Source: Front Public Health. 2023 Nov 29;11:1280941. doi: 10.3389/fpubh.2023.1280941 (PMC10724027; doi:10.3389/fpubh.2023.1280941)
Supplement: Supplementary file 1 [file Data_Sheet_1.docx]

# Appendix 1: interview guide

**The significance of an active healthy lifestyle**

**In-depth Interviews**

**Objective:**

Understand the target populations needs and motivations regarding physical activity supported by technology and 2) define the problem regarding the lack of physical activity,

*The interview is being recorded and all data will be processed anonymously.*

**Defining terms**

Physical Activity = "This is an activity in which you consciously engage in additional physical activity (movement) that accelerates your breathing and/or your heart rate. For example, walking, swimming, yoga, other exercises or sports."

**Demographic information**

- What is your age?
- You are: Male / Female
- What is your highest degree of education?
  - Primary school
  - Secondary school / high school
  - Higher education: bachelor (graduate)
  - University: bachelor/master
  - Post-university: doctorate
- Are you retired? Yes / No
  - If no: Are you currently engaged in paid activity?
- In which sector have you worked or are you currently working? Or what was your profession?
- Are you a caregiver? Yes / No
- Do you receive care? Yes / No (= someone who cares for you with whom you have a personal relationship)
- Where do you live? - Please provide your postal code
- What is your marital status? Married / Cohabiting / Widow/Widower / Divorced / Single
- Who do you live with? o I live alone o I live with my partner o I live with my children and/or grandchildren o Other, namely:...
- What is your type of residence? o House with garden or terrace o Apartment with garden or terrace o Kangaroo home o Group home o Service flat / assisted living facility o Nursing home
- To what extent are sports facilities near your residence? Consider parks, sports halls, swimming pools, forests, fitness trails, etc. o Within 1km o Between 1-10 km o More than 10 km
- Are they accessible to you? o On foot o By bike o By mobility scooter / electric wheelchair o By car o By public transportation
- What transportation options do you have? (multiple answers are possible) o Bicycle (non-electric) o Electric bicycle o Mobility scooter / electric wheelchair o Car o Bus o Train o Taxi o Other, namely:
- Do you still drive a car yourself? o Yes o No
- Who takes you to your destinations? o My partner o My children o My grandchildren o My neighbor o Other, namely: ...
